# Interprofessional Collaboration—Time for a New Theory of Action?

**DOI:** 10.3389/fmed.2022.876715

**Published:** 2022-03-18

**Authors:** Ray Samuriwo

**Affiliations:** Faculty of Health Studies, School of Nursing and Healthcare Leadership, University of Bradford, Bradford, United Kingdom

**Keywords:** interprofessional collaboration, systems thinking, muddy zone of practice, theory of action, healthcare professions education

## Introduction

Interprofessional collaboration (IPC) is integral to the quality, equity, justice, and safety of healthcare (1–3). Having a diverse group of healthcare professionals engaged in IPC with different backgrounds, insights and perspectives increases the chances of generating unique and innovative solutions to challenges that often arise with regards to care quality in clinical practice. However, there is a long history of shortcomings in IPC that have a deleterious impact on patient safety arising from conflict relating to professional boundaries, license, jurisdiction, and mandate between different healthcare professionals, such as doctors and nurses (4–14). These recurring narratives about the relationships between doctors and nurses, who are in two of the oldest healthcare professions, highlight the challenges that exist in facilitating IPC which achieves the lofty aim of consistently delivering safe, high-quality care to all in a just and equitable manner. Efforts to improve IPC have mainly relied on interprofessional education, learning, or leadership interventions to foster a collegiate and integrated approach to the healthcare in which the contribution of people from different disciplines is valued (15–18). The success of IPC improvement efforts based on interprofessional education, learning, and leadership has been mixed (18–23). The reported variation in the efficacy of different interprofessional education and learning efforts in bringing about IPC may be due to the focus on teaching and upskilling individuals, groups, or teams from different professions with the objective of making them more collaborative. It is worth considering the nature of context in which IPC takes place and some of the factors that are at play which may account of the mixed results of improvement efforts.

## Healthcare Systems, and IPC

Healthcare is delivered in a pressurized context with a complex adaptive ecology by systems which are inherently fractal and self-similar (24–27). There are also a wide range of psychological, social and individual “human factors” that are at play within complex healthcare systems that influence healthcare professionals' clinical practice and determine the quality of patient care (28–30). Healthcare systems are the product of socio-cultural beliefs, norms, and value ecologies that interact in complex ways, but are manifested explicitly, or tacitly in the behavior of individual actors. Consequently, there are many factors that affect healthcare professionals IPC in clinical practice, which means that IPC improvement efforts need to be cognizant of individual, social and cultural factors that arise due the course of care delivery in different contexts. There are benefits in using a systems-thinking approach to consider why they continue to be many reported challenges relating to IPC and care quality in clinical practice. Systems-thinking or the capacity to analyze systems in their totality is of cardinal importance in healthcare, which is delivered in systems which are complex and concatenated (31–33). Over the last decade, there has been a move toward integrating systems-thinking into different facets of healthcare professional education with varying levels of success (31–33). Some of the more recent efforts to integrate systems thinking into medical education have extended to interprofessional education and IPC (34, 35). Efforts to extend systems thinking into healthcare professional education and IPC have at times received a lukewarm reception because of uncertainty about its application, and a mistaken view that it is peripheral to clinical practice (34, 35). Even though there appear to be challenges with regards to the adoption of systems-thinking, it is worth considering how it can inform new ways of thinking about IPC and how it can be addressed.

## IPC as a Muddy Zone of Practice

There are many as aspects of healthcare professional education that are laden with complexity, contingencies, uncertainties, and unintended consequences that are often referred to as “*muddy zones of practice*” (36). Interprofessional collaboration is in many respects a muddy zone of practice in healthcare professional education, which is often cited as a causative or contributory factor to adverse patient safety events and near misses (1–3). The recurring narrative relating to interprofessional collaboration manifests a key characteristic of muddy zones of practice, which often appear to be intractable or resistant to improvement initiatives (36). Veen and Canciolo (36) contend that addressing the complex problems that constitute muddy zones of practice in healthcare professional education require a slow, deliberate, and considered approach which reconsiders prevailing practice in an effort to get a better perspective of the situation in which things are seen more clearly, and can be done in better or more appropriate ways. This exhortation suggests that there is value in a systems-thinking approach which conceptualizes IPC as a muddy zone of practice and considering what can be done to address its concomitant challenges with regards to healthcare professional education and patient care in clinical practice.

## Toward a New Theory of Action

Given the complex adaptive ecology of healthcare systems, and the plethora of human factors that arise in clinical practice, understanding IPC as a muddy zone of practice points to improvement efforts with a different theory of action. Healthcare is delivered in systems that are incessantly evolving to populations with values, norms and expectations that are constantly shifting. The organization and delivery of healthcare is reliant on healthcare professionals with their own values, beliefs and attitudes which are moderated and influenced by a variety of different socio-cultural factors. In addition, healthcare professionals often belong to a discipline specific community of practice with its own distinct professional identity, license, jurisdiction, and mandate that may be contested by others and give rise to conflict that undermines IPC. Catastrophic failures in healthcare often arise in organizations and systems where there is a dominant culture or mind-set which overlooks alternatives that are inconsistent with the dominant group narrative (37, 38). Considering the environment in which these failures arise, IPC needs to function and be effective in systems where healthcare professionals' practice which is subject to and influenced by the prevailing culture. The culture in any facet of healthcare invariably has people that are consigned or ascribed to in groups, out groups or a subculture (39). Continuing with a systems-thinking mindset and understanding IPC as a muddy zone of practice, a different view of healthcare professionals with the same nature, beliefs, and socio-cultural influences as any other human being points toward a more nuanced theoretically informed approach.

Meaningful change arises when things are understood as they are experienced, and people have a theory of action that reflects their reality and praxis (40). There may be scope then, to develop IPC improvement interventions that better reflect the complex and evolving nature of healthcare. Modern healthcare is not just about treating a condition or managing an illness, but it is about providing people with the treatment that they need and providing them with the knowledge and support that they need to live healthy and fulfilling lives. Given the rapid changes that can arise in societal norms, culture, and the health of populations as evinced by the COVID-19 pandemic; there are many challenges that lie ahead to facilitate IPC that enhances the quality of care. The COVID-19 pandemic has also surfaced the impact that faith and belief have on how people act and behave in relation to healthcare. In Theaetetus, Plato defined knowledge as the intersection of truth and belief where knowledge cannot be claimed if something is true but not believed, or believed but not true (41–43). This assertion gives added credence to the notion that efforts to understand and improve IPC must reflect the reality of patient care as experienced by healthcare professionals so that they not only see and understand its relevance but believe that they have a key role to play in it as part of their responsibility to improve patient care.

In sum, it may be prudent to focus on ensuring that the theory of action that underpins efforts to embed and improve IPC in clinical reflects the vicissitudes of patient care is credible and is believed by the healthcare professionals whose practice it seeks to change. Efforts to improve and embed IPC that enhances patient safety, requires healthcare professionals with an appropriate mindset, skills, attitude, as well as insight or belief to interpret and utilize the evidence at hand appropriately to improve the health and wellbeing of those in their ward with due consideration of their values or preferences.

## Conclusion

Improving IPC and the quality of healthcare has long been the focus of considerable improvement efforts with varying levels of success. In more recent times, there has been a better understanding of the complexity of healthcare systems and their impact on the behavior and actions of healthcare professionals. Systems-thinking is a useful way of understanding the nature of healthcare systems and designing improvement interventions that reflect the complex ecology of organizational and human factors in clinical practice. While efforts to improve IPC using approaches informed by systems-thinking have limited success thus far, there is still merit and scope in pursuing this line of endeavor. Reconceptualizing IPC as a muddy zone of practice that requires improvement is consistent with a systems-thinking approach, but points toward a more nuanced theory of action to underpin improvement efforts. Such as theory of action needs to reflect the reality of healthcare professionals from different backgrounds if the objective of improving the quality of patient care is to be achieved. Education fulfills its true emancipatory apogee, or pinnacle, when students and educators collectively develop a dialogical theory of praxis as a community (44). If the quality, safety, justice, and equity of healthcare is to be improved through IPC, then it would be apt for healthcare professions educators to focus on creating IPC landscapes of practice with communities of healthcare professionals, educators, students from different disciplines engaged in an ongoing dialogue and working partnership in which everyone is heard, seen, and valued for their contribution to healthcare. Perhaps then, a shared dialogical theory of meaning and action aligned to a culture of effective IPC can be fostered and flourish within the complex milieu of health care systems. Thus, IPC may one day cease to be a muddy zone of practice in healthcare professionals' education.

## Author Contributions

The author confirms being the sole contributor of this work and has approved it for publication.

## Conflict of Interest

The author declares that the research was conducted in the absence of any commercial or financial relationships that could be construed as a potential conflict of interest.

## Publisher's Note

All claims expressed in this article are solely those of the authors and do not necessarily represent those of their affiliated organizations, or those of the publisher, the editors and the reviewers. Any product that may be evaluated in this article, or claim that may be made by its manufacturer, is not guaranteed or endorsed by the publisher.

## References

[1] Jeffs L Abramovich IA Hayes C Smith O Tregunno D Chan W-H. Implementing an interprofessional patient safety learning initiative: insights from participants, project leads and steering committee members. BMJ Qual Safety. (2013) 22:923–30. 10.1136/bmjqs-2012-001720 23771901

[2] Reeves S Pelone F Harrison R Goldman J Zwarenstein M. Interprofessional collaboration to improve professional practice and healthcare outcomes. Cochrane Database Syst Rev. (2017) 6:CD000072. 10.1002/14651858.CD000072.pub3 28639262 PMC6481564

[3] Petit dit Dariel O Cristofalo P. A meta-ethnographic review of interprofessional teamwork in hospitals: what it is and why it doesn't happen more often. J Health Serv Res Policy. (2018) 23:272–9. 10.1177/1355819618788384 30033767

[4] Stein LI. The doctor-nurse game. Arch Gen Psychiatry. (1967) 16:699–703. 10.1001/archpsyc.1967.01730240055009 6027368

[5] Muir J. Nurses respond to doctor-nurse game. AORN J. (1978) 28:653. 10.1016/S0001-2092(07)61674-3 251076

[6] Stein LI Watts DT Howell T. The doctor–nurse game revisited. N Engl J Med. (1990) 322:546–9. 10.1056/NEJM199002223220810 2300124

[7] Sweet SJ Norman IJ. The nurse-doctor relationship: a selective literature review. J Adv Nurs. (1995) 22:165–70. 10.1046/j.1365-2648.1995.22010165.x 7560525

[8] Willis E Parish K. Managing the doctor-nurse game: a nursing and social science analysis. Contemp Nurse. (1997) 6:136–44. 10.5172/conu.1997.6.3-4.136 9511655

[9] Radcliffe M. Doctors and nurses: new game, same result. BMJ. (2000) 320:1085. 10.1136/bmj.320.7241.1085 10764392 PMC1117967

[10] Gjerberg E Kjolsrod L. The doctor-nurse relationship: how easy is it to be a female doctor co-operating with a female nurse? Soc Sci Med. (2001) 52:189–202. 10.1016/S0277-9536(00)00219-7 11144775

[11] Reeves S Nelson S Zwarenstein M. The doctor-nurse game in the age of interprofessional care: a view from Canada. Nurs Inquiry. (2008) 15:1–2. 10.1111/j.1440-1800.2008.00396.x 18271784

[12] Holyoake DD. Is the doctor-nurse game being played? Nurs Times. (2011) 107:12–4. 23251967

[13] Whitehead B. Nurses should defy the deeply embedded doctor-nurse game. Nurs Times. (2011) 107:11. 23251966

[14] Mertens F De Gendt A Deveugele M Van Hecke A Pype P. Interprofessional collaboration within fluid teams: community nurses' experiences with palliative home care. J Clin Nurs. (2019) 28:3680–90. 10.1111/jocn.14969 31216390

[15] WHO. Framework for Action on Interprofessional Education & Collaborative Practice. Geneva: World Health Organisation (2010). 21174039

[16] IOM. Interprofessional Education for Collaboration: Learning How to Improve Health from Interprofessional Models Across the Continuum of Education to Practice: Workshop Summary. Washington, DC: Institute of Medicine and the National Academies Press (2013). 24901189

[17] Wakefield A Carlisle C Hall A Attree M. Patient safety investigations: the need for interprofessional learning. Learn Health Soc Care. (2009) 8:22–32. 10.1111/j.1473-6861.2008.00192.x

[18] Braithwaite J Westbrook M Nugus P Greenfield D Travaglia J Runciman WB. Continuing differences between health professions' attitudes: the saga of accomplishing systems-wide interprofessionalism. Int J Qual Health Care. (2013) 25:8–15. 10.1093/intqhc/mzs071 23203766

[19] Cresswell K Howe A Steven A Smith P Ashcroft D Fairhurst K. Patient safety in healthcare preregistration educational curricula: multiple case study-based investigations of eight medicine, nursing, pharmacy and physiotherapy university courses. BMJ Qual Saf . (2013) 22:843–54. 10.1136/bmjqs-2013-001905 23728120

[20] Brock D Abu-Rish E Chiu C-R Hammer D Wilson S Vorvick L. Interprofessional education in team communication: working together to improve patient safety. BMJ Qual Saf . (2013) 22:414–23. 10.1136/bmjqs-2012-000952 23293118

[21] Wang Z Feng F Gao S Yang J. A systematic meta-analysis of the effect of interprofessional education on health professions students' attitudes. J Dent Educ. (2019) 83:1361–9. 10.21815/JDE.019.147 31548305

[22] van Duin TS de Carvalho Filho MA Pype PF Borgmann S Olovsson MH Jaarsma ADC. Junior doctors' experiences with interprofessional collaboration: wandering the landscape. Med Educ. (2022) 56:418–31. 10.1111/medu.14711 34890487 PMC9305225

[23] Young J Zolio L Brock T Harrison J Hodgkinson M Kumar A. Interprofessional learning about medication safety. Clin Teach. (2021) 18:656–61. 10.1111/tct.13430 34669259

[24] Plsek PE Greenhalgh T. The challenge of complexity in health care. BMJ. (2001) 323:625–8. 10.1136/bmj.323.7313.625 11557716 PMC1121189

[25] Pattison S Samuriwo R. Ceeing compassion in care: more than ‘Six C'S'? Nurs Philos. (2016) 17:140–3. 10.1111/nup.12110 26676742

[26] Braithwaite J. Changing how we think about healthcare improvement. BMJ. (2018) 361:k2014. 10.1136/bmj.k2014 29773537 PMC5956926

[27] Sturmberg JP Martin CM. Universal health care - a matter of design and agency? J Eval Clin Pract. (2021) 27:1011–7. 10.1111/jep.13395 32267086

[28] WHO. Human factors in patient safety. Review of topics and tools. Report for methods and measures working group of WHO Patient Safety. Geneva: World Health Organisation (2009).

[29] Russ AL Fairbanks RJ Karsh B-T Militello LG Saleem JJ Wears RL. The science of human factors: separating fact from fiction. BMJ Qual Saf . (2013) 22:802–8. 10.1136/bmjqs-2012-001450 23592760 PMC3786617

[30] Xie A Carayon P. A systematic review of human factors and ergonomics (HFE)-based healthcare system redesign for quality of care and patient safety. Ergonomics. (2015) 58:33–49. 10.1080/00140139.2014.959070 25323570 PMC4297241

[31] Colbert CY Ogden PE Ownby AR Bowe C. Systems-based practice in graduate medical education: systems thinking as the missing foundational construct. Teach Learn Med. (2011) 23:179–85. 10.1080/10401334.2011.561758 21516607

[32] Obeso VT Phillipi CA Degnon CA Carter TJ. A systems-based approach to curriculum development and assessment of core entrustable professional activities in undergraduate medical education. Med Sci Educ. (2018) 28:407–16. 10.1007/s40670-018-0540-7

[33] Woodruff JN. Accounting for complexity in medical education: a model of adaptive behaviour in medicine. Med Educ. (2019) 53:861–73. 10.1111/medu.13905 31106901

[34] Gonzalo JD Davis C Thompson BM Haidet P. Unpacking medical students' mixed engagement in health systems science education. Teach Learn Med. (2019) 32:250–8. 10.1080/10401334.2019.1704765 31875724

[35] Gonzalo JD Chang A Dekhtyar M Starr SR Holmboe E Wolpaw DR. Health systems science in medical education: unifying the components to catalyze transformation. Acad Med. (2020) 95:1362–72. 10.1097/ACM.0000000000003400 32287080

[36] Veen M Cianciolo AT. Problems no one looked for: philosophical expeditions into medical education. Teach Learn Med. (2020) 32:337–44. 10.1080/10401334.2020.1748634 32272853

[37] Ravaghi H Mannion R Sajadi HS. Organizational failure in an NHS hospital trust: a qualitative study. Health Care Manager. (2015) 34:367–75. 10.1097/HCM.0000000000000087 26506299

[38] Kirkup B. The Report of the Morecambe Bay Investigation. An independent investigation into the management, delivery and outcomes of care provided by the maternity and neonatal services at the University Hospitals of Morecambe Bay NHS Foundation Trust from January 2004 to June 2013. London: The Stationery Office (2015).

[39] Samuriwo R. Values, culture, narrative and medical education: the case for a renewed focus. Med Educ. (2021) 55:888–9. 10.1111/medu.14569 34003497

[40] Rosmini A. Principles of Ethics. Translated by Denis Cleary and Terence Watson. Edited by Antonio Belsito. Durham: Rosmini Publications (1988).

[41] Cooper JM. Plato's Theaetetus. London; New York, NY: Routledge (1990).

[42] Jowett B. The Dialogues of Plato. Translated into English with analyses and introductions in five volumes. Vol. IV. 3rd ed. London: Oxford University Press (1892).

[43] Campbell L. The Theaetetus of Plato. A Revised Text and English Notes. 2nd ed. London: Oxford University Press (1883).

[44] Samuriwo R Patel Y Bullock A. Response to: ‘Gender bias in medical education: stop treating it as an inevitability'. Med Educ. (2020) 54:864. 10.1111/medu.14205 32350894

